# Detection and Classification of Unhealthy Heartbeats Using Deep Learning Techniques

**DOI:** 10.3390/s25195976

**Published:** 2025-09-26

**Authors:** Abdullah M. Albarrak, Raneem Alharbi, Ibrahim A. Ibrahim

**Affiliations:** Computer Science Department, College of Computer and Information Sciences, Imam Mohammad Ibn Saud Islamic University (IMSIU), Riyadh 11432, Saudi Arabia; amsbarrak@imamu.edu.sa (A.M.A.);

**Keywords:** deep learning, ECG, arrhythmia, time series, multi-model, classification, discrete wavelet transform, grey wolf optimizer, AAMI

## Abstract

Arrhythmias are a common and potentially life-threatening category of cardiac disorders, making accurate and early detection crucial for improving clinical outcomes. Electrocardiograms are widely used to monitor heart rhythms, yet their manual interpretation remains prone to inconsistencies due to the complexity of the signals. This research investigates the effectiveness of machine learning and deep learning techniques for automated arrhythmia classification using ECG signals from the MIT-BIH dataset. We compared Gradient Boosting Machine (GBM) and Multilayer Perceptron (MLP) as traditional machine learning models with a hybrid deep learning model combining one-dimensional convolutional neural networks (1D-CNNs) and long short-term memory (LSTM) networks. Furthermore, the Grey Wolf Optimizer (GWO) was utilized to automatically optimize the hyperparameters of the 1D-CNN-LSTM model, enhancing its performance. Experimental results show that the proposed 1D-CNN-LSTM model achieved the highest accuracy of 97%, outperforming both classical machine learning and other deep learning baselines. The classification report and confusion matrix confirm the model’s robustness in identifying various arrhythmia types. These findings emphasize the possible benefits of integrating metaheuristic optimization with hybrid deep learning.

## 1. Introduction

Cardiac arrhythmia represents a growing global health problem characterized by irregularities in heart rhythm that can range from benign to life-threatening. Accurate detection and classification of arrhythmias remain a persistent challenge, particularly due to the limitations of conventional diagnostic techniques in handling subtle and complex variations in cardiac electrical activity [1]. As heart disease rates increase worldwide, mainly due to demographic aging and modifiable lifestyle risks, the need for precise and prompt arrhythmia detection has grown significantly. Electrocardiography (ECG) is the primary non-invasive tool used to monitor and evaluate heart rhythm abnormalities [2]. However, traditional ECG interpretation is often hampered by poor signal quality, transient anomalies, and the subtle manifestation of certain arrhythmic patterns [3]. Moreover, accurate analysis requires significant clinical expertise, and manual interpretation remains labor-intensive and susceptible to fatigue-induced errors [4]. These challenges emphasize the need to develop advanced computational methods that can improve diagnostic precision and support clinicians in managing the growing volume and complexity of arrhythmia cases.

In the last decade, artificial intelligence (AI) has revolutionized healthcare, especially in diagnostic imaging and signal processing [2]. Machine learning (ML) and deep learning (DL) algorithms detect patterns in data, outperform conventional statistical approaches, and enhance diagnostic accuracy [5]. Decision trees, support vector machines, and ensemble models have been widely applied in ECG signal analysis, based on characteristics such as peak detection and heart rate variability [6]. However, their performance is restricted by feature selection, making feature engineering a critical yet limiting phase [7].

DL models differ from traditional ML models in that they learn hierarchical representations directly from raw data, without manual feature extraction [8]. Interpretation of the ECG is further hampered by patient variation, such as age, sex, and BMI, which influence signal stability [7]. Movement noise, electrode displacement, and electrical artifacts also complicate interpretation [9]. Visual inspection similarly introduces variability, as physicians may reach different conclusions on the same ECG, leading to misdiagnosis [10]. Advanced cases require highly specialized expertise, increasing the likelihood of diagnostic errors [11]. One-dimensional convolutional neural networks (1D-CNNs) and long short-term memory networks (LSTMs) are commonly applied in ECG analysis: 1D-CNNs extract spatial patterns and help identify abnormalities such as myocardial infarcts and arrhythmias, while LSTMs, with their memory cells, capture long-term dependencies in ECG signals [12,13]. Recent developments have brought hybrid models that integrate 1D-CNNs for feature extraction and LSTMs for temporal analysis, enhancing classification accuracy and robustness against noise and missing data [14,15].

Despite these developments, DL techniques remain computationally intensive and require large amounts of data. Limited datasets restrict the real-world application of deep learning models in small, resource-limited clinical settings [16,17]. Moreover, these models often lack interpretability, which is essential for clinical adoption, regulatory approval, and overall trust [18]. Recent studies have attempted to address these challenges. For example, a 1D-CNN method achieved approximately 98% precision in classifying heart disease, while hybrid models based on ensemble methods and RNNs improved precision but demanded more computational resources due to their complexity [19,20]. Other research employed CNN-GRU and SIFT-based CNN models using spectrograms, demonstrating superior performance but also increased computational complexity [21].

This research uses the Grey Wolf Optimizer (GWO), a metaheuristic optimization algorithm, to further enhance the performance and adaptability of multi-model deep learning models by tuning the hyperparameters of the hybrid 1D-CNN-LSTM architecture. The GWO, inspired by the leadership hierarchy and hunting behavior of gray wolves in nature, has proven effective in balancing exploration and exploitation in search spaces. By automatically optimizing hyperparameter configurations, the GWO improves model accuracy while reducing manual tuning efforts, contributing to better generalization and computational efficiency [22].

Therefore, the primary gap identified in existing research is that deep learning methods face limitations in widespread adoption within small-scale clinical settings due to their high complexity and low interpretability. This study proposes a new deep learning model for analyzing ECG signals and compares it with other models in terms of complexity, interpretability, and predictive precision. The goal is to improve computational efficiency and enhance the explainability of the results, contributing to the development of more accurate and clinically applicable diagnostic models, and supporting medical decision-making through advanced diagnostic systems.

This work aims to advance computer-automated models of accurate ECG interpretation by examining ML and DL techniques based on their complexity, explainability, and predictive capabilities. Consistent with the identified research gaps, this study addresses limitations of current models, such as high computational complexity, the requirement for large datasets, and poor interpretability. This research, therefore, aims to bridge these gaps by presenting solutions that maintain high accuracy while improving computational efficiency and clinical interpretability, leading to more practical and reliable ECG diagnostic tools for healthcare applications.

### 1.1. Challenges and Gaps

Traditional methods for ECG analysis face several significant challenges. Variations in patient physiology can cause substantial differences in ECG readings, even among patients with similar health profiles. In addition, noise from sources such as patient movement, electrode misplacement, or electrical interference can obscure critical ECG features, thereby affecting the accuracy of diagnosis. Visual interpretation of ECG also presents challenges; inconsistencies can arise between observers or even within the same observer over time, leading to missed or incorrect diagnoses. Moreover, complex arrhythmias often require specialized expertise, increasing the probability of diagnostic errors in routine practice.

AI techniques offer the potential for more accurate and automated arrhythmia detection and classification, yet they also face obstacles. Ensuring high precision, managing imbalanced data, effectively tuning hyperparameters, and handling noise are key challenges that must be addressed to improve the reliability and performance of arrhythmia detection systems. In particular, deep learning models are highly sensitive to the choice of hyperparameters, and suboptimal tuning can significantly degrade their predictive accuracy and generalization. Thus, optimizing these parameters becomes a critical step in achieving efficient and robust performance.

### 1.2. Motivation

The diagnosis of arrhythmias using ECG data requires expert analysis and a deep understanding of heart patterns. Although experienced cardiologists can identify arrhythmias through manual ECG examinations, there is a risk that less experienced practitioners may misinterpret the signals, leading to inaccurate diagnoses. This issue is critical given the life-threatening nature of heart arrhythmias, which reinforces the importance of timely and accurate diagnosis.

This highlights the importance of improving automated systems for the detection and classification of arrhythmias, especially considering the challenges faced by current machine learning or deep learning methods, such as long training periods, computational complexity, and manual feature engineering or parameter tuning. To address these challenges, this research aims to develop a proposed multi-model system that integrates deep learning techniques, such as 1D-CNN and LSTM networks. These models have demonstrated strong capabilities in learning spatial and temporal patterns from raw ECG signals.

Moreover, to further enhance the proposed multi-model system, this research introduces a metaheuristic optimization strategy, the GWO, to automatically tune the hyperparameters of the constituent models. This biologically inspired algorithm mimics the leadership hierarchy and hunting behavior of gray wolves in nature, and it has shown strong performance in global optimization tasks. By embedding the GWO into the multi-model training pipeline, the system can achieve better convergence, reduce overfitting risks, and maximize classification accuracy, especially when dealing with diverse arrhythmia patterns.

It is also important to evaluate performance beyond simple accuracy, considering other metrics such as precision, recall, and the F1 score, which offer a more comprehensive assessment of the effectiveness of the model in handling various classes of arrhythmias. Finally, models that perform well under experimental conditions must be tested in real-world environments to account for the variability and additional challenges that clinical practice may present.

### 1.3. Objectives

The goal of this research is to create and implement a multi-model system for detecting and classifying arrhythmias using advanced DL methods, specifically 1D-CNN and LSTM. We aim to accomplish the following specific objectives:

- Enhance the classification accuracy of arrhythmia types by developing a multi-model system specifically designed for ECG-based detection.
- Integrate a GWO algorithm to automatically optimize the hyperparameters of the multi-model DL framework, enhancing performance and reducing manual trial-and-error tuning.
- Evaluate the proposed multi-model against traditional classifiers, such as the Gradient Boosting Model (GBM) and Multilayer Perceptron (MLP), in terms of predictive performance and computational efficiency.
- Compare individual DL models (e.g., 1D-CNN and LSTM) with the proposed optimized multi-model framework to emphasize the benefits of integration and metaheuristic-based optimization.

This work aims to develop models for accurate and automated ECG interpretation to support clinical diagnosis. It explores the strengths and limitations of ML and DL models, focusing on factors such as complexity, interpretability, and predictive accuracy. The findings will advance intelligent healthcare systems, improving patient outcomes, while demonstrating how DL models, when combined with optimization algorithms such as GWO, can enhance precision and clinical efficiency.

The next sections of this work will provide a literature review, dataset description, data preprocessing, methodology, experiments, and results, and the final section will outline conclusions and future work. This structured approach guarantees the investigation of all aspects of AI’s application and improvement in detecting and classifying arrhythmias through ECG analysis.

## 2. Literature Review

Recent research has extensively explored automated arrhythmia detection using ECG signals, particularly through DL and hybrid techniques to improve diagnostic precision and generalization. Many studies have introduced innovative architectures and preprocessing methods to address common challenges in ECG analysis and improve overall performance.

Mj Alqaisi et al. [23] proposed a hybrid framework combining 1D-CNN, LSTM networks, and the Discrete Wavelet Transform (DWT). Their model achieved 97% accuracy on the MIT-BIH dataset, highlighting the potential of combining spatial and temporal features. However, their study was limited to four heartbeat types, reducing its applicability to broader classification tasks.

Similarly, Shoughi et al. [24] developed a CNN–Bidirectional LSTM framework incorporating the DWT for denoising and SMOTE for class balancing, reaching 98% accuracy in five AAMI categories. While impressive, this approach relied heavily on oversampling and did not address hyperparameter tuning, which may impact robustness.

Hassan et al. [25] integrated CNN and B-LSTM layers for arrhythmia detection across the MIT-BIH and St. Petersburg datasets. Despite achieving 98% on MIT-BIH, accuracy declined on the external dataset, exposing limitations in cross-dataset generalization.

Other studies have explored image-based or mobile-focused approaches. For instance, Prusty et al. [21] proposed a scale-invariant CNN using SIFT for ECG image classification, achieving 99.78% accuracy. While highly accurate, their method relied on image transformation, which may lose the temporal dynamics inherent in ECG signals. Similarly, Eleyan et al. [19] introduced Rhythmi, a mobile CNN-based tool trained on PhysioNet datasets, showing real-time feasibility. In further work, they converted ECG time series into spectrograms with hybrid feature extraction, but the added complexity makes real-world deployment more challenging.

Optimization-based methods have also gained attention. RCNN-GWO et al. [26] optimized a Recurrent CNN with the GWO, achieving 98% on the MIT-BIH and PTB datasets. However, their work focused on RCNN, leaving the potential of CNN-LSTM unexplored. Likewise, Mishra et al. [27] applied GWO to an ANN model, achieving a modest accuracy of 93.38%, showing that GWO can enhance classical models but remains limited compared to deep hybrids.

A review of previous studies shows significant advances in the detection and classification of arrhythmias using DL techniques. These methods have shown excellent performance in identifying patterns in ECG data. However, many of these studies focus on a limited scope, often classifying only a few types of arrhythmias, and may not fully utilize the combined strengths of different architectures for feature extraction and sequential pattern analysis in ECG data.

In contrast, this research proposes a new approach that integrates feature extraction and temporal dependency analysis into a unified model. Specifically, the proposed model includes a nine-layer feature extraction process to capture critical patterns in ECG signals, followed by temporal analysis to explore long-term relationships within the data. The final classification process aims to detect five arrhythmias based on the standards of the Association for the Advancement of Medical Instrumentation (AAMI), addressing challenges related to feature extraction and sequential patterns in ECG data. To further enhance performance, a GWO is used to fine-tune the model’s hyperparameters.

## 3. Dataset Description

The ECG data used in this study were collected from the MIT-BIH Arrhythmia Database, a widely utilized repository of ECG recordings in the cardiology domain [28]. This database consists of 48 half-hour segments of two-lead ambulatory ECG from 47 patients recorded between 1975 and 1979: 25 males and 22 females, aged 23 to 89. The recordings were sampled at 360 Hz, digitized with a resolution of 11 bits over a 10 mV range, and include reference annotations for more than 110,000 heartbeats [29].

**Figure 1:**
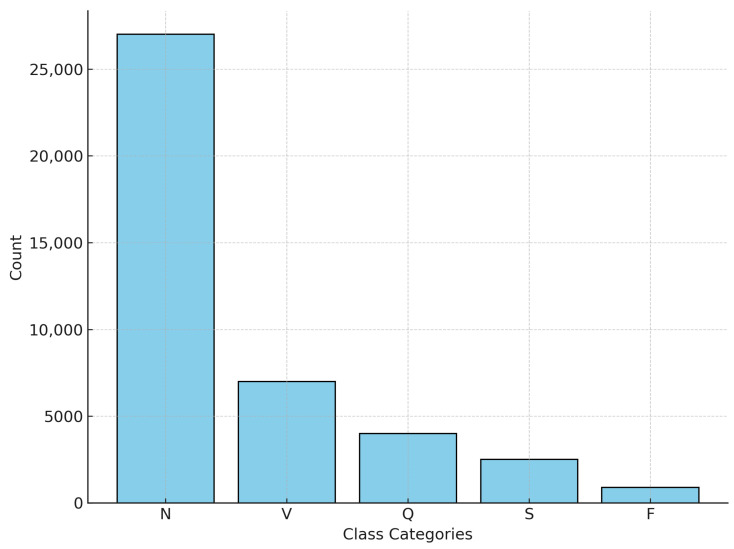
Distribution of arrhythmia classes in the MIT-BIH dataset.

These annotations label various arrhythmic and normal beats, which were consolidated into five target classes based on the standards of AAMI, as shown in Table 1 [30]. The dataset contains a total of 44,408 samples, of which 33,370 were allocated for training and 8,343 for testing. An important challenge is the class imbalance problem: the normal class represents the majority of the available samples, while other arrhythmia classes are significantly underrepresented, as illustrated in Figure 1.

## 4. Methodology

**Figure 2:**
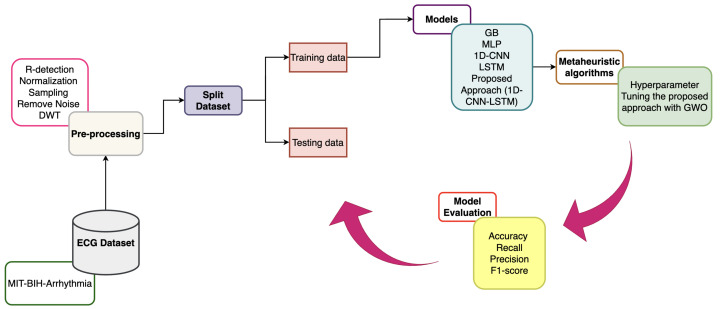
Proposed arrhythmia diagnosis methodology.

The proposed approach to the diagnosis of arrhythmias, illustrated in Figure 2, follows a structured process to improve the accuracy of arrhythmia detection and classification. The process begins with preprocessing ECG signals from the MIT-BIH Arrhythmia Database, which includes R-peak detection, normalization, sampling, noise removal, and the DWT to ensure high-quality input data. After preprocessing, the dataset is divided into training and testing sets for model evaluation.

This approach utilizes a 1D-CNN as a feature extractor to identify significant spatial patterns in ECG signals, while an LSTM network captures crucial temporal dependencies to distinguish between different types of arrhythmia. The proposed 1D-CNN-LSTM model integrates these two architectures, allowing the CNN to extract spatial features while LSTM processes sequential patterns, thereby improving classification performance.

To further enhance the model’s effectiveness, a metaheuristic optimization technique, the GWO, is employed to automatically fine-tune the hyperparameters of the proposed hybrid model. By mimicking the leadership hierarchy and hunting behavior of gray wolves in nature, the GWO improves the search for optimal parameter configurations, reducing the need for manual tuning and contributing to better performance.

Additionally, to benchmark its effectiveness, the proposed model is compared against traditional ML algorithms, including GBM and MLP models, as well as single DL models such as 1D-CNN and LSTM. The evaluation uses performance metrics such as accuracy, precision, recall, and the F1 score, providing a comprehensive assessment of the model’s robustness in the classification of arrhythmias.

### 4.1. Preprocessing Pipeline

**Figure 3:**
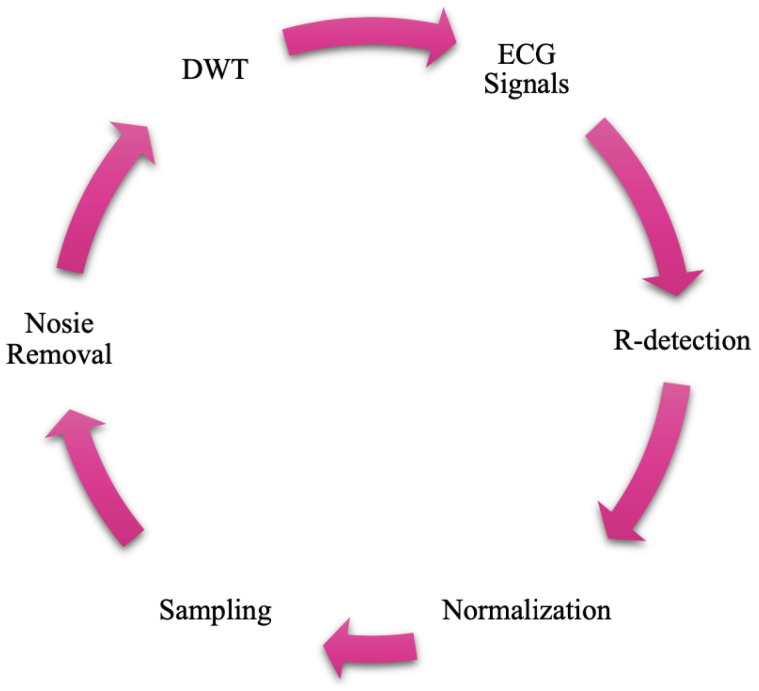
Preprocessing pipeline for ECG signals.

Preprocessing plays a crucial role in accurately detecting arrhythmias during ECG signal analysis. Figure 3 illustrates the preprocessing pipeline applied to the ECG signals prior to classification. The process begins with the acquisition of raw ECG signals, followed by R-peak detection to accurately segment individual heartbeats. Normalization is then applied to standardize amplitude variations across signals, ensuring consistency. A sampling step is introduced not only to extract fixed-length heartbeat segments but also to address the issue of data imbalance across different arrhythmia classes, thereby ensuring a more balanced representation of samples. Subsequently, the DWT is applied to remove baseline wander and high-frequency noise. This sequence of steps enhances signal quality and preserves the morphological and temporal features essential for reliable arrhythmia classification.

#### 4.1.1. ECG Signals (Features and Variations)

**Figure 4:**
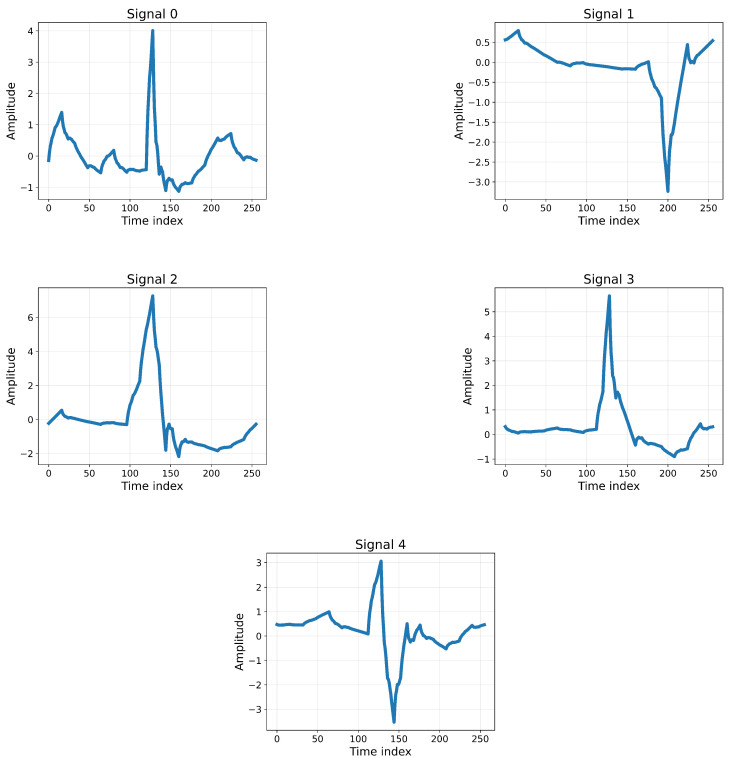
Representative ECG signal samples from the MIT-BIH dataset.

Figure 4 presents representative ECG signal samples extracted from the MIT-BIH dataset, each illustrating different morphological patterns of cardiac activity. The plots capture key waveform components such as the P-wave, QRS complex, and T-wave, which are critical indicators for arrhythmia classification. The diversity in amplitude and shape across samples highlights the variability in patient heart rhythms, demonstrating the challenges of distinguishing between normal and abnormal beats. By showcasing these variations, the figure provides an analytical view of the input data fed into the proposed models, emphasizing the necessity of robust feature extraction through 1D-CNN layers and temporal pattern learning via LSTM. These signal samples not only contextualize the dataset but also underscore the importance of preprocessing and class balancing steps in preparing the data for accurate classification.

In particular, we can observe the essential features of the ECG signals: the types of beats and the quality of the signal. The x-axis corresponds to the time index (signal time points), while the y-axis represents the amplitude of the ECG signal. A closer look at the samples reveals distinct characteristics: Sample 0 appears regular with a clearly distinguishable P wave, QRS complex, and T wave; Sample 1 is also near-regular but shows slight abnormalities; Sample 2 demonstrates minor deviations but remains close to a normal baseline; Sample 3 exhibits stronger fluctuations and noticeable differences, especially in the ST segment, suggesting possible abnormality; and Sample 4 shows a less distinct QRS complex with a prolonged QT interval, which may indicate underlying cardiac conditions. These illustrative variations highlight the physiological complexity of ECG signals and justify the use of advanced deep learning methods for reliable arrhythmia detection.

#### 4.1.2. R-Peak Detection

R-peak detection is a crucial step in ECG signal processing, especially in heartbeat analysis. The RS wave is part of the QRS complex and is the most recognizable wave in an ECG signal, indicating ventricular depolarization [31]. The detection of R-waves is necessary for numerous applications, including heart rate monitoring, arrhythmia identification, and feature selection for further analysis [32]. The ECG signals are divided into segments to identify their start and end points, which helps determine the R-peak value for each signal. Accurate identification of R-peaks is essential for calculating heart rate variability and diagnosing various types of arrhythmias and other cardiac conditions.

#### 4.1.3. Normalization

Normalization is a critical preprocessing step for ECG signals, aligning their values within a specified range to improve the accuracy and stability of the model [33]. This process involves calculating the mean and standard deviation of the signal, adjusting it to have a mean of 0 and a standard deviation of 1, and finally scaling it to a consistent amplitude range. This ensures improved accuracy and minimizes potential distortions.

#### 4.1.4. Sampling

To address the class imbalance in the ECG dataset, a selective sampling approach was applied. The process involves reducing the overrepresentation of the ’normal’ class signals in the dataset. Specifically, for each signal annotation, the algorithm checks whether the signal belongs to a predefined class. If the signal is classified as ’normal,’ it is only retained with a probability of 0.15. This strategy ensures that only a subset of normal signals is included, thereby creating a more balanced dataset across all classes. This targeted sampling method makes the overall distribution of signals more representative, which is crucial for effectively training models.

#### 4.1.5. Noise Removal

In clinical environments, ECG signals are often corrupted by noise due to factors such as patient movement, electrical interference, or electrode displacement [34]. Therefore, a systematic approach to noise removal was applied. Techniques such as DWT filtering were employed to eliminate unwanted frequencies while preserving the relevant ECG signal components. In addition, NaN or infinite values in the dataset were carefully handled to avoid errors during analysis and ensure smooth processing.

#### 4.1.6. Discrete Wavelet Transform (DWT)

The DWT is well suited for processing ECG signals due to its ability to simultaneously analyze both time and frequency, which is critical for handling non-stationary signals such as ECG [35]. The DWT provides multi-resolution analysis that decomposes the signal into separate frequency bands, enabling a detailed examination of its components.

The DWT improves signal quality by effectively reducing noise while preserving essential features, such as R-peaks, through the application of high-pass and low-pass filters. The high-pass filter captures high-frequency details, while the low-pass filter focuses on low-frequency components. This process, repeated across multiple levels, generates wavelet coefficients that represent both approximations and details of the signal, thereby reducing its length and optimizing it for subsequent analysis [36].

A key step in the DWT application is selecting the mother wavelet, which determines the decomposition’s effectiveness. Commonly used wavelets include Daubechies, Symlets, and Coiflets, each chosen according to specific requirements such as smoothing or feature extraction. In this study, the Symlet5 (sym5) wavelet was selected, with decomposition performed at three levels to ensure effective noise reduction and feature retention [37].

To denoise the signal, thresholding was applied to the wavelet coefficients. Donoho’s method was used to compute the noise threshold (λ) based on the Mean Absolute Deviation (MAD) of the detail coefficients [38]. The equations for estimating the noise level (σ) and threshold (λ) are as follows:

(1) $$\begin{array}{cc} \sigma & =\frac{\mathrm{MAD}(|cd|)}{0.6745} \end{array}$$

(2) $$\begin{array}{cc} \lambda & =\sigma \sqrt{2\log(n)} \end{array}$$

**Figure 5:**
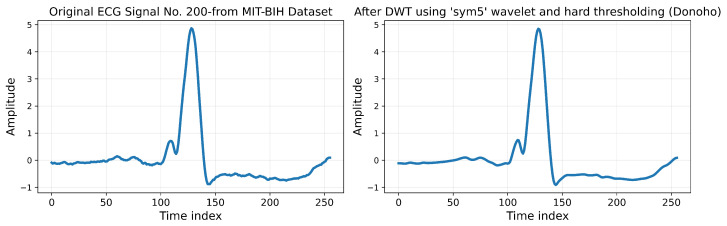
Example of ECG signal No. 200: Original vs. denoised version after DWT.

Here, σ represents the estimated noise level, cd denotes the detail coefficients, and *n* is the length of the signal. A hard thresholding operation is applied to suppress noise components, while approximation coefficients are retained. The denoised signal is then reconstructed using the inverse DWT. The processed ECG signals after applying the DWT are shown in Figure 5, which clearly demonstrates the preservation of essential ECG features, such as R-peaks, while reducing unwanted noise.

#### 4.1.7. Dataset Split

As shown in Table 2, the MIT-BIH dataset was divided into training and testing sets using an 80/20 ratio, ensuring proportional representation across all five arrhythmia classes. This division produced 33,436 training samples and 8360 testing samples, covering N, S, V, F, and Q categories. Such a split maintains balance across classes, allowing for a reliable evaluation of the proposed model.

### 4.2. One-Dimensional Convolutional Neural Network Model (1D-CNN)

The 1D-CNN, which utilizes convolution and pooling layers, aims to identify characteristics indicative of different aspects of heart behavior. Thus, it is suitable for the detection and classification of arrhythmias [39]. The input data are processed through filters in the convolutional layer that identify patterns and shapes at different levels of abstraction to extract meaningful features.

**Figure 6:**
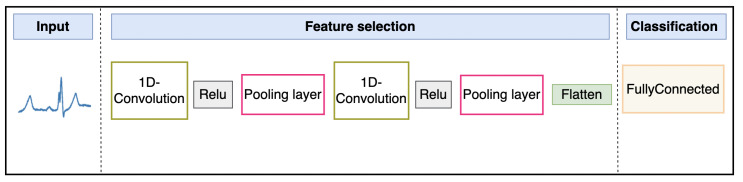
Architecture of 1D-CNN.

Meanwhile, the feature maps obtained from the convolutional layers apply an activation function called the Rectified Linear Unit (ReLU). This function introduces non-linearity, allowing the network to learn more complex representations of the data [40]. The pooling layer downsamples the feature maps of the previous layers, reducing the spatial dimensions without losing significant features. This process improves computational efficiency as it extracts relevant details from the data. The block diagram of the convolutional neural network is shown in Figure 6.

The 1D-CNN architecture consists of two convolutional layers, each followed by a ReLU activation function, dropout layers to prevent overfitting, and max-pooling layers that reduce the dimensionality of the data while preserving important features. The first convolutional layer applies 256 filters with a kernel size of 8 and a stride of 5, followed by a max-pooling operation that halves the data size. The second convolutional layer uses 128 filters with a kernel size of 7 and a stride of 4, again followed by max-pooling, as shown in Table 3. After the convolution and pooling operations, the output is flattened and passed through a fully connected layer to produce a final feature vector, which is then fed into the classifier.

#### 1D-CNN Implementation

The 1D-CNN model was implemented in PyTorch version 2.8.0 and trained on GPU: Tesla T4, Memory: 14.74 GB. The training was performed for 30 epochs with a batch size of 20. The Adam optimizer was employed with a learning rate of 0.00015. To mitigate class imbalance, a weighted cross-entropy loss function was used. During training, accuracy and loss were monitored on both training and testing sets across all epochs to ensure stable convergence.

### 4.3. Long Short-Term Memory Model (LSTM)

**Figure 7:**
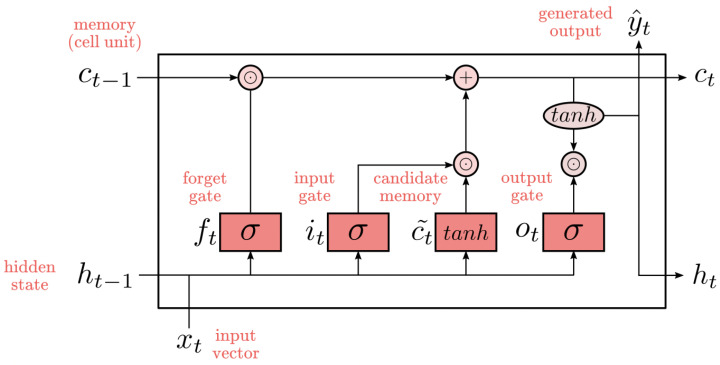
LSTM architecture.

The LSTM algorithm is notable for its ability to automatically extract features from ECG data and learn complex non-linear relationships. Therefore, the LSTM model is frequently used for feature extraction and classification of non-linear signals such as ECG, EMG, and EEG [41]. The representation of the LSTM architecture, with its blocks and memory cell units, is shown in Figure 7. LSTM offers a significant advantage through its internal components, known as “gates”, which are crucial in managing the flow of information within the network. These gates, including the input gate, forget gate, change gate, and output gate, allow precise control over the addition and removal of information within the cell. This capability enables the cell to retain specific values over defined time intervals. The input gate governs the timing of new information entering memory, the forget gate controls the removal of outdated information, and the output gate determines when the stored information should be used in the cell output [42].

In an LSTM cell, two distinct states exist: the short-term ($h_{t}$) and the long-term ($C_{t}$) cell states. These states are essential for the intermediate processing of information. LSTM cells also include four trainable gates: input, output, forget, and update. These gates regulate the flow of information, determining which data is added or discarded, thus allowing the cell to retain information for specific time intervals. Functioning as neural networks, the gates control what information can enter or exit the cell state. They also determine, during training, which data should be retained or forgotten within the cell’s memory.

The input data at time *t* is represented as $x=\{x_{1},x_{2},\ldots ,x_{n}\}$. The cell’s memory ($C_{t}$) is updated using the input ($i_{t}$), forget ($f_{t}$), and change ($C_{t}$) gates. The operations performed by these gates at any given time *t* are as follows:

(3) $$\begin{array}{cc} i_{t} & =\sigma (W_{i}x_{t}+W_{hi}h_{t-1}+b_{i}) \end{array}$$

(4) $$\begin{array}{cc} f_{t} & =\sigma (W_{f}x_{t}+W_{fi}h_{t-1}+b_{f}) \end{array}$$

(5) $$\begin{array}{cc} o_{t} & =\sigma (W_{o}x_{t}+W_{oi}h_{t-1}+b_{o}) \end{array}$$

(6) $$\begin{array}{cc} C_{t}^{∼} & =\tanh(W_{c}x_{t}+W_{hc}h_{t-1}+b_{c}) \end{array}$$

(7) $$\begin{array}{cc} C_{t} & =f_{t}⊙C_{t}+i_{t}⊙C_{t}^{∼} \end{array}$$

(8) $$\begin{array}{cc} h_{t} & =o_{t}⊙\tanh\left(C_{t}\right) \end{array}$$

Here, σ represents the sigmoid activation function and tanh represents the hyperbolic tangent activation function. The matrices and vectors of weights—$W_{hc},W_{hi},W_{f},W_{fi},W_{o},$$W_{oi},W_{c},W_{i}$—along with the bias vectors ($b_{i},b_{f},b_{o},b_{c}$), determine the operation of the gates. The ⊙ symbol indicates element-wise multiplication. The gates operate similarly to traffic controllers, regulating the movement of information through the cell state and ensuring that only relevant data is passed through. The input gate updates the cell’s memory based on the previous memory block and the tanh function, while the output gate produces the final output of the current LSTM block.

The LSTM architecture consists of three layers with decreasing hidden sizes: the first layer has 64 units, the second 32, and the third 16. A dropout rate of 0.4 is applied between the layers to prevent overfitting, followed by a fully connected layer. The model output is classified using a Softmax function, as shown in Table 4. This design enables the model to capture both short-term and long-term dependencies in ECG signals, improving its ability to detect arrhythmias.

#### LSTM Implementation

The LSTM model was implemented in PyTorch version 2.8.0 and trained on GPU: Tesla T4, Memory: 14.74 GB. The training was performed for 30 epochs with a batch size of 20. The Adam optimizer was used with a learning rate of 0.0002, while a weighted cross-entropy loss function was applied to mitigate class imbalance. Throughout training, both accuracy and loss were monitored on the training and testing sets to ensure stable convergence.

### 4.4. Machine Learning Models (ML)

Traditional ML models applied to the same preprocessed MIT-BIH arrhythmia dataset serve as baselines to evaluate the performance of the proposed DL models in the classification of arrhythmias. GBMs are employed as powerful ensemble techniques that combine multiple weak learners, typically decision trees, to form a strong predictive model. GBMs work by sequentially training models, where each model corrects the errors of its predecessor, thus improving classification accuracy over iterations. MLP networks, a type of artificial neural network, are used to classify arrhythmias through a simple feedforward network, serving as a baseline to compare the effectiveness of shallow networks versus more complex DL models.

### 4.5. 1D-CNN-LSTM Model (Proposed Approach)

**Figure 8:**
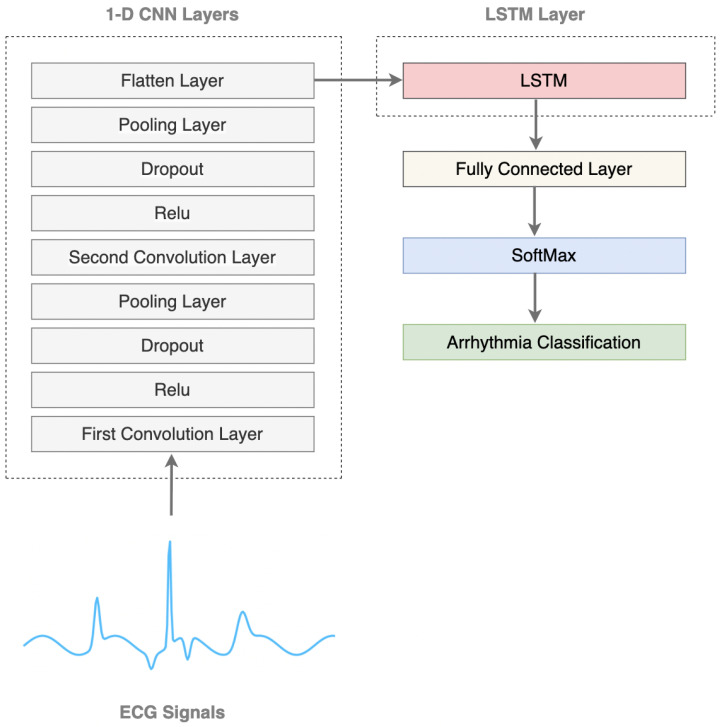
Proposed approach 1D-CNN-LSTM model architecture.

A novel DL system is proposed for the detection and classification of arrhythmia by integrating the components of the 1D-CNN and the LSTM, as shown in the system architecture in Figure 8. The ECG data from the MIT-BIH arrhythmia dataset is initially processed through a 1D-CNN model that comprises a sequence of convolutional layers followed by ReLU activation functions, pooling layers, and a flattening layer. To prevent overfitting, dropout layers are added after the activation function.

Specifically, the 1D-CNN architecture consists of multiple convolutional layers with ReLU activation functions and pooling operations to extract hierarchical characteristics from the input ECG data, along with a flattening layer that transforms multidimensional input data into a one-dimensional vector, allowing the network to learn hierarchical features effectively [43]. The extracted feature vectors refer to the transformed representations of the input data obtained in multiple layers of the 1D-CNN. These characteristic vectors consist of all the weights in the 1D-CNN and capture essential patterns and information present in the input ECG data. Therefore, extracted features are fed into an LSTM layer, allowing the model to capture temporal dependencies and long-term patterns in the ECG data. Subsequently, the LSTM output is passed through a fully connected layer to learn the relationships between the extracted features and make predictions.

Finally, a Softmax activation function is applied to the output of the fully connected layer to produce the final classification results for the detection of arrhythmias. This comprehensive approach utilizes the strengths of the 1D-CNN in feature extraction and LSTM in sequence modeling to enhance the precision and efficiency of the ECG classification task.

### 4.6. Grey Wolf Optimizer for Hyperparameter Selection

To further enhance the classification performance of the proposed 1D-CNN-LSTM hybrid model, this research incorporates a metaheuristic optimization technique known as the Grey Wolf Optimizer (GWO). The primary goal of integrating the GWO is to automatically fine-tune the hyperparameters of the model, such as the number of filters, kernel size, batch size, learning rate, number of LSTM units, and dropout rate. Manual tuning of these parameters is often time-consuming and suboptimal, whereas the GWO provides an efficient global search mechanism to explore the parameter space.

#### 4.6.1. Overview of GWO

The GWO is a population-based metaheuristic inspired by the leadership hierarchy and cooperative hunting strategies of gray wolves in nature [22]. The algorithm classifies the population of candidate solutions into four hierarchical categories:

- Alpha (α): Represents the best candidate solution.
- Beta (β): Represents the second-best solution.
- Delta (δ): Represents the third-best solution.
- Omega (ω): Represents the remaining candidate solutions.

**Figure 9:**
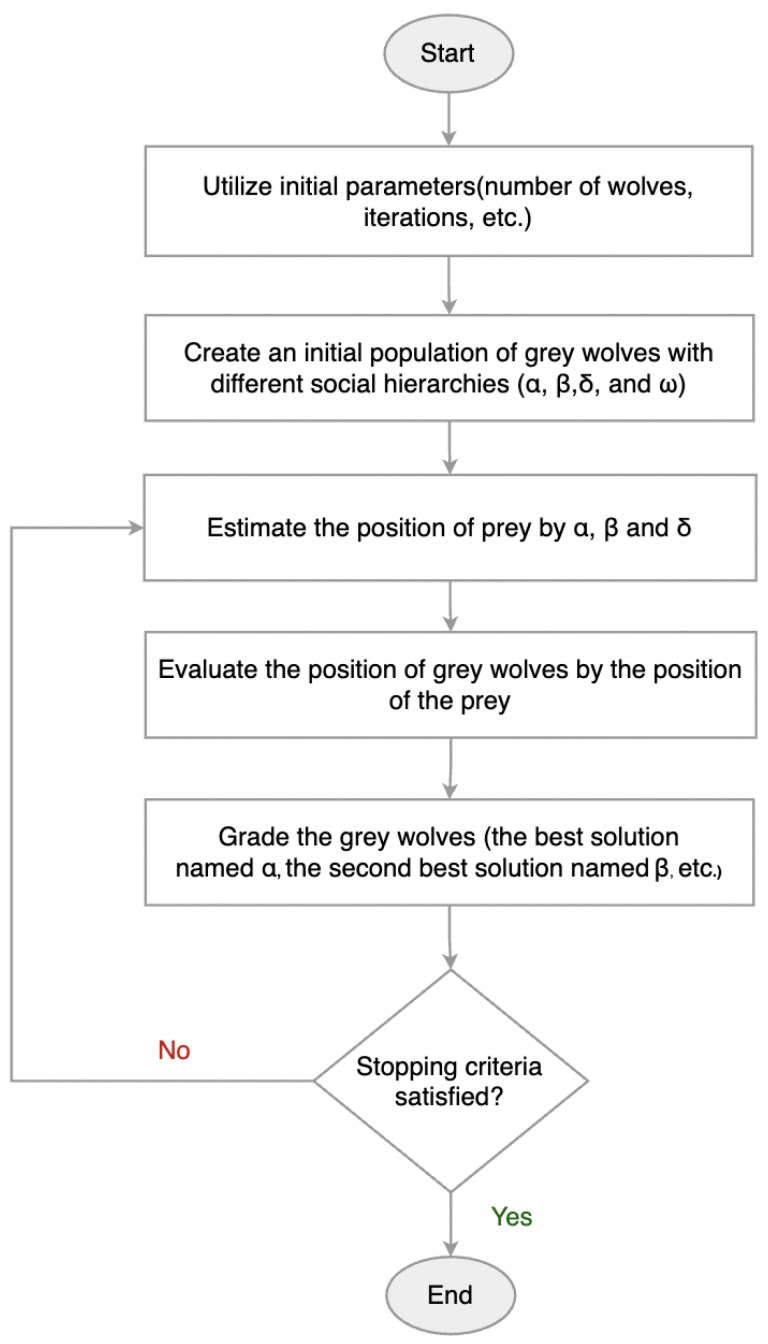
Flowchart illustrating the main steps of the GWO algorithm.

These wolves collaborate to update their positions and gradually converge toward the global optimum by mimicking the processes of encircling, hunting, and attacking prey. The overall workflow of the GWO is visually summarized in Figure 9, while its procedural steps are presented as pseudocode in Algorithm 1. This combination provides a clear conceptual and procedural understanding before delving into the mathematical formulation of the algorithm.

**Algorithm 1:** GWO pseudocode.

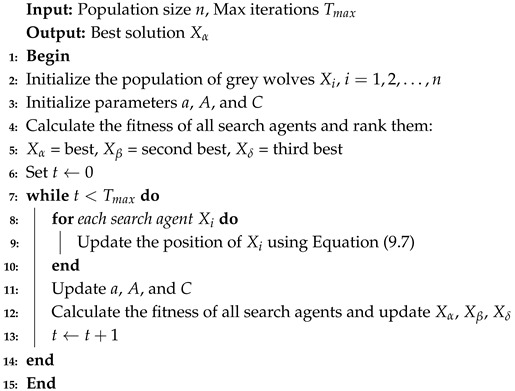



#### 4.6.2. Mathematical Modeling of GWO

The position of a gray wolf (candidate solution) is updated based on the positions of α, β, and δ wolves. The encircling behavior is mathematically modeled as follows:

(9) $$\vec{D}=\left|\vec{C}\cdot {\vec{X}}_{p}\left(t\right)-\vec{X}\left(t\right)\right|,$$

(10) $$\vec{X}\left(t+1\right)={\vec{X}}_{p}\left(t\right)-\vec{A}\cdot \vec{D},$$

where

- ${\vec{X}}_{p}$ is the position of the prey (best solution);
- $\vec{X}$ is the current position of the gray wolf;
- $\vec{A}$ and $\vec{C}$ are coefficient vectors calculated as follows:

(11) $$\vec{A}=2\vec{a}\cdot {\vec{r}}_{1}-\vec{a},\;\vec{C}=2\cdot {\vec{r}}_{2},$$

where ${\vec{r}}_{1}$, ${\vec{r}}_{2}$ are random vectors in [0, 1], and $\vec{a}$ linearly decreases from 2 to 0 over the course of iterations.

Each candidate solution’s position is influenced by the top three wolves (α, β, δ) as follows:

(12) $${\vec{X}}_{1}={\vec{X}}_{\alpha }-{\vec{A}}_{1}\cdot \left|{\vec{C}}_{1}\cdot {\vec{X}}_{\alpha }-\vec{X}\right|,$$

(13) $${\vec{X}}_{2}={\vec{X}}_{\beta }-{\vec{A}}_{2}\cdot \left|{\vec{C}}_{2}\cdot {\vec{X}}_{\beta }-\vec{X}\right|,$$

(14) $${\vec{X}}_{3}={\vec{X}}_{\delta }-{\vec{A}}_{3}\cdot \left|{\vec{C}}_{3}\cdot {\vec{X}}_{\delta }-\vec{X}\right|,$$

(15) $$\vec{X}\left(t+1\right)=\frac{{\vec{X}}_{1}+{\vec{X}}_{2}+{\vec{X}}_{3}}{3}.$$

This update mechanism ensures exploration in the early iterations and exploitation as the algorithm converges.

#### 4.6.3. Hyperparameter Space and Initialization

To maintain realistic and efficient optimization, the GWO algorithm explores the search space within manually defined ranges for each hyperparameter, ensuring values remain within practical limits. The ranges listed in Table 5 are utilized as follows.

Each candidate solution is initialized with random values within these ranges and evaluated based on a fitness function, defined as the classification accuracy on the validation set.

#### 4.6.4. Fitness Function and Optimization Process

The fitness function *F* used by the GWO is defined as follows:

(16) $$F={\mathrm{Accuracy}}_{val}\left(\vec{X}\right),$$

where $\vec{X}$ represents a candidate hyperparameter set. During each iteration, the population is updated according to the GWO update rules, and the best solution (i.e., highest validation accuracy) is selected as the optimal hyperparameter set for the final model.

#### 4.6.5. Proposed Approach Implementation

The proposed approach model (1D-CNN–LSTM) was implemented in PyTorch, combining convolutional layers for feature extraction with an LSTM layer to capture temporal dependencies in ECG sequences. To avoid manual trial-and-error tuning, the GWO was employed to automatically optimize the key hyperparameters, including convolutional filter sizes, stride lengths, dropout rates, pooling parameters, and LSTM hidden units. Training was performed using the AdamW optimizer with a learning rate of 0.001 and weighted cross-entropy loss to address class imbalance. The optimization was conducted with a population of 10 wolves over 20 iterations, and the best-performing configuration was selected based on validation accuracy.

### 4.7. Evaluation Metrics

Customized evaluation metrics are used to assess the performance of the models in arrhythmia detection, ensuring that the specific characteristics of the dataset and task are appropriately captured. Given the critical nature of arrhythmias, these metrics evaluate the models’ ability to recognize different types while minimizing both false positives and false negatives.

(17) $$\begin{array}{cc} \;\mathrm{Overall}\;\mathrm{Accuracy} & =\frac{\mathrm{TP}+\mathrm{TN}}{\mathrm{TP}+\mathrm{TN}+\mathrm{FP}+\mathrm{FN}}\times 100 \end{array}$$

(18) $$\begin{array}{cc} \mathrm{Recall} & =\frac{\mathrm{TP}}{\mathrm{TP}+\mathrm{FN}} \end{array}$$

(19) $$\begin{array}{cc} \mathrm{Precision} & =\frac{\mathrm{TP}}{\mathrm{TP}+\mathrm{FP}} \end{array}$$

(20) $$\begin{array}{cc} F1\text{-}\mathrm{score} & =2\times \frac{\mathrm{Recall}\times \mathrm{Precision}}{\mathrm{Recall}+\mathrm{Precision}} \end{array}$$

In these equations, T, F, P, and N denote true, false, positive, and negative, respectively. For example, TP refers to the number of true positives, while FN refers to the number of false negatives.

## 5. Results and Discussion

The MIT-BIH Arrhythmia Database was used for this research, which contains approximately 110,000 heartbeat annotations collected from 48 half-hour ECG recordings of 47 subjects. The signals were sampled at 360 Hz with 11-bit resolution over a 10 mV range, ensuring high-quality recordings suitable for detecting subtle arrhythmia patterns. A subset of these signals was carefully selected to ensure a balanced representation of the five arrhythmia classes, as defined by the AAMI standard.

Various ML and DL models were applied to classify these ECG signals, including MLP, GBM, 1D-CNN, LSTM, and the proposed approach 1D-CNN–LSTM. Each model’s capability to capture spatial and temporal patterns in ECG signals was evaluated, ensuring a comprehensive comparison of their classification performance. The results, detailing accuracy and efficiency differences among these models, are summarized in Table 6.

### 5.1. ML Models

**Figure 10:**
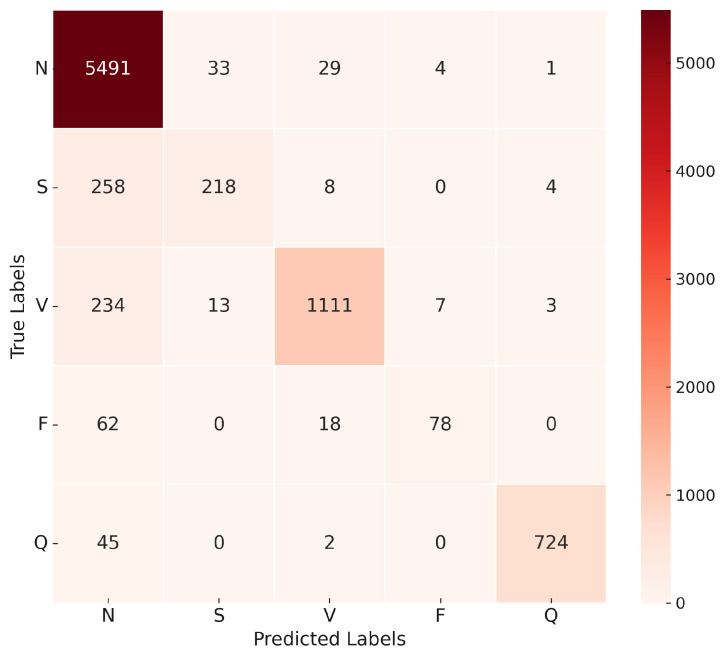
Confusion matrix for GBM.

In this experiment, the GBM was trained to iteratively improve classification performance by combining multiple weak learners, typically decision trees, into a strong ensemble. The model was configured using default hyperparameters, which were empirically validated to provide reliable performance on the dataset. The GBM achieved an overall accuracy of 91%, which is considered moderately acceptable; however, it was not among the top-performing models. Given the nature of ECG signals, where diagnostic accuracy is critical, higher performance is expected to ensure clinical reliability. Due to the sequential nature of boosting, training time may increase with larger datasets. As shown in Figure 10, the confusion matrix for the GBM highlights its classification accuracy across different classes, with notable strengths in well-represented categories and some misclassifications in borderline or underrepresented classes. As detailed in the evaluation matrix shown in Table 7, the model exhibited high precision and recall for the N and Q classes, while the S and F classes showed moderate performance, suggesting areas for further optimization.

**Figure 11:**
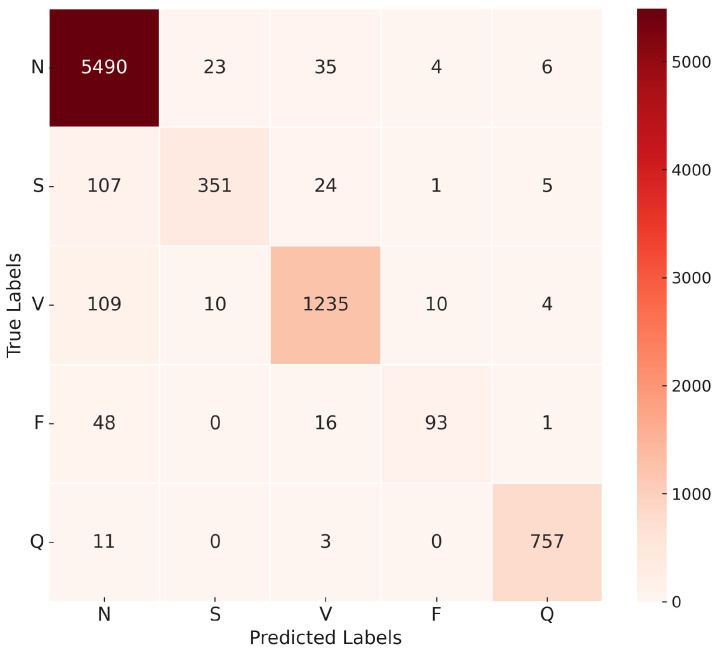
Confusion matrix for MLP model.

The MLP model, a type of artificial neural network, was configured with one hidden layer of 10 neurons and utilized the ReLU activation function to introduce non-linearity. The model was trained using the Adam optimizer, which dynamically adjusts learning rates during training. With a regularization term α=0.0004 to mitigate overfitting, the MLP achieved an overall accuracy of 95%. As shown in Figure 11, the confusion matrix reveals that the MLP model performed well overall but exhibited slight misclassifications in certain classes, particularly the F and S categories. As shown in Table 8, the evaluation matrix corroborates these findings, showing that while the N and Q classes achieved high precision and recall, other classes require improved tuning to enhance model performance.

The confusion matrices and classification reports for both the GBM and MLP models demonstrate their respective strengths and areas for improvement. The GBM achieved an overall accuracy of 91%, while the MLP performed slightly better with an overall accuracy of 95%. These findings underscore the importance of understanding the nuances of each model, including the impact of hyperparameter selection and data preprocessing, to achieve optimal results in arrhythmia classification. Given the critical nature of ECG data, models with higher precision and consistency are favored to ensure reliable diagnostic support.

### 5.2. DL Models

**Figure 12:**
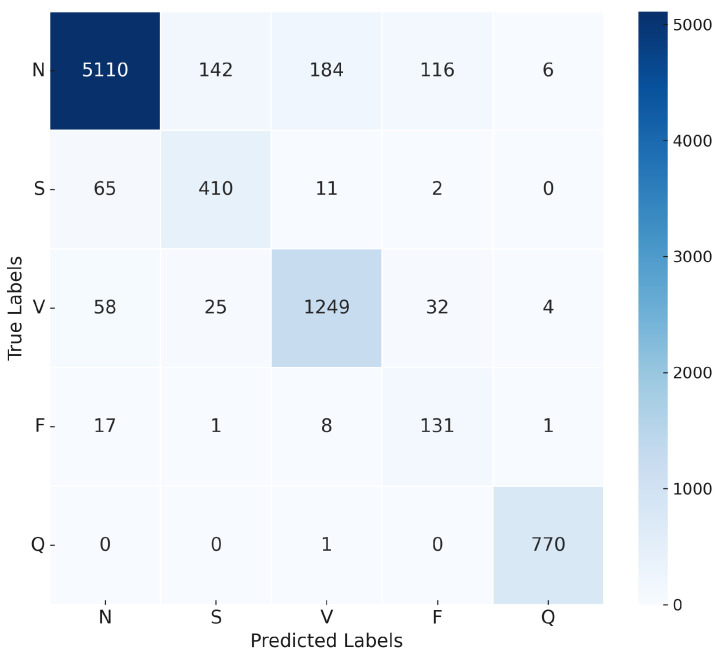
Confusion matrix for 1D-CNN model.

**Figure 13:**
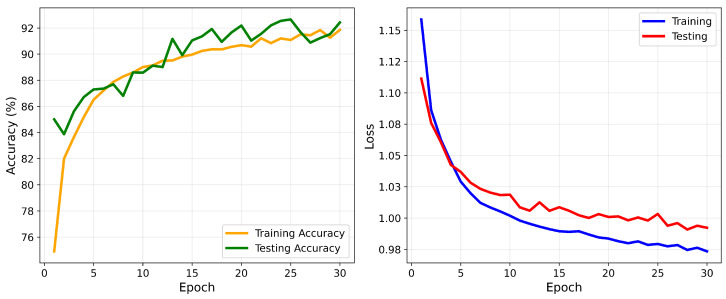
Accuracy and loss convergence for the 1D-CNN model.

The 1D-CNN was designed to extract local features from ECG signals through convolutional layers. The model was trained with a learning rate of 0.0002 and the Adam optimizer. The CrossEntropyLoss function was employed, and class weights were applied to address the class imbalance. After training with a batch size of 20, the model achieved an overall accuracy of 92% and demonstrated strong performance across most classes. For class N, it achieved a precision of 97% and a recall of 92%, indicating high precision and effective detection. However, challenges were noted for class F, which had a precision of 47%, suggesting room for improvement. As shown in Figure 12, the confusion matrix highlights the model’s classification accuracy for each class. Table 9 presents the evaluation matrix, revealing an F1 score of 95% for class N and 89% for class V, reflecting robust detection capabilities. However, the F1 score for class F was lower at 60%, underscoring difficulties in this category. These results highlight the model’s strengths in accurately classifying common classes but also suggest opportunities to refine its performance for less distinct categories, such as class F. Adjustments to hyperparameters or increased model depth may address these challenges and improve its classification capabilities. Figure 13 illustrates the training and validation accuracy and loss curves for the 1D-CNN model across 30 epochs.

**Figure 14:**
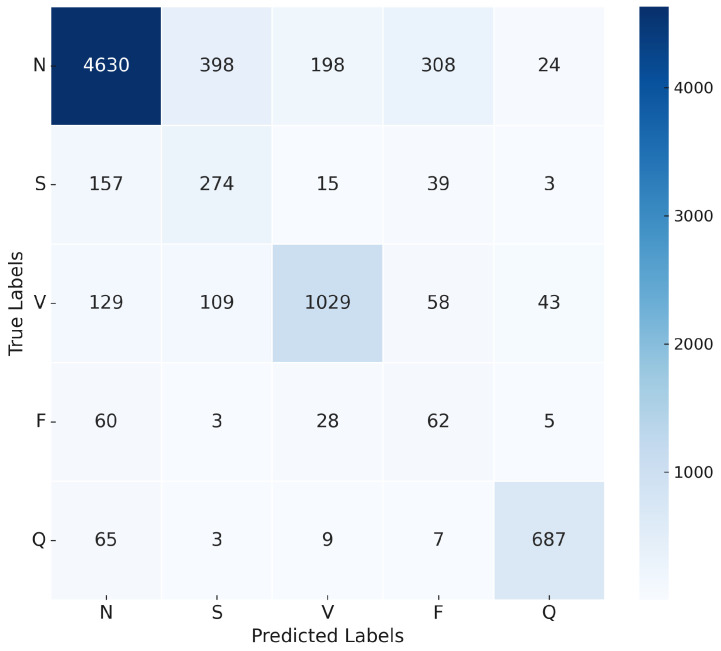
Confusion matrix for LSTM model.

**Figure 15:**
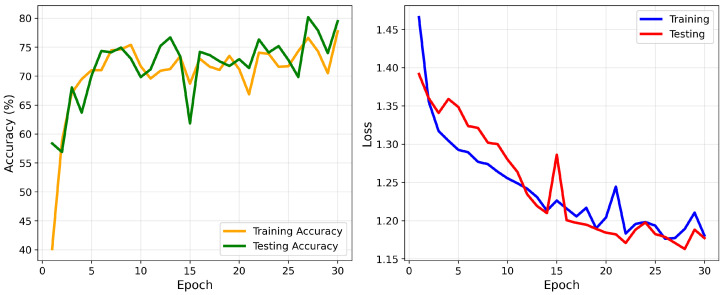
Accuracy and loss convergence for the LSTM model.

The LSTM model was implemented to capture the temporal dependencies in ECG signals, which are crucial for arrhythmia classification. Similarly to the 1D-CNN model, the Adam optimizer was used with a learning rate of 0.0002. The model was trained with a batch size of 16, and CrossEntropyLoss was applied. The LSTM model, while effective at capturing temporal patterns, achieved a slightly lower overall accuracy of 75%, likely due to its inability to capture local features, unlike the 1D-CNN. As shown in Figure 14, the confusion matrix highlights the model’s classification accuracy for each class. The LSTM model shows strong performance in the N and Q classes, achieving high precision and recall metrics. However, it struggles with the S and F classes, as indicated by low precision (35% and 13%, respectively) and moderate recall (56% and 39%, respectively), as shown in Table 10. These results suggest misclassification issues due to unclear features or insufficient data. The training and validation accuracy and loss curves for the LSTM model over 30 epochs are shown in Figure 15.

The proposed approach model combines the strengths of both 1D-CNN and LSTM networks, where the 1D-CNN component is responsible for extracting local features from the ECG signals, while the LSTM component captures temporal dependencies within the sequences. The model architecture is based on a 1D-CNN-LSTM hybrid structure.

To optimize the model’s performance, the GWO algorithm was employed to automatically select the best hyperparameters, such as the number of filters in each 1D-CNN layer, kernel sizes, dropout rates, pooling configurations, and LSTM hidden size. This approach replaced the manual tuning process, enabling a more systematic and efficient exploration of the hyperparameter space to enhance model accuracy.

For weight initialization, the Kaiming Normal initialization method was used to ensure efficient gradient flow during training. This technique, also known as He initialization [44], is particularly suited for layers followed by ReLU activations and helps prevent vanishing or exploding gradients in deep networks. It was applied to all 1D-CNN, linear, and LSTM layers in the model to stabilize training and improve convergence.

**Figure 16:**
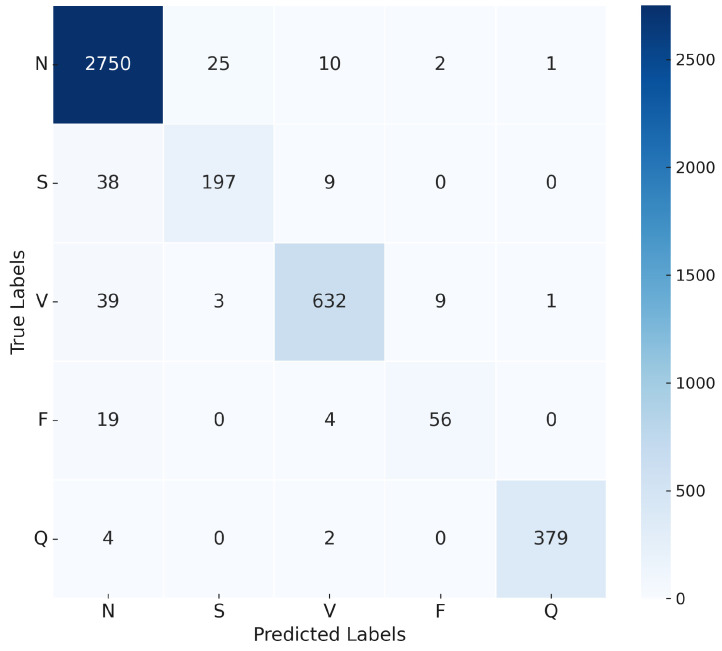
Confusion matrix for the proposed approach 1D-CNN-LSTM model.

The confusion matrix in Figure 16 illustrates the model’s ability to distinguish between the five classes of ECG recommended by AAMI. The matrix shows a high true positive rate for the N and V classes, with minor misclassifications occurring in the S and F classes.

The evaluation matrix presented in Table 11 further supports the effectiveness of the model, with a weighted average precision, recall, and F1 score. Although the model achieved outstanding results in most categories, it showed a relatively lower recall in the F class, suggesting potential areas for future improvement.

**Figure 17:**
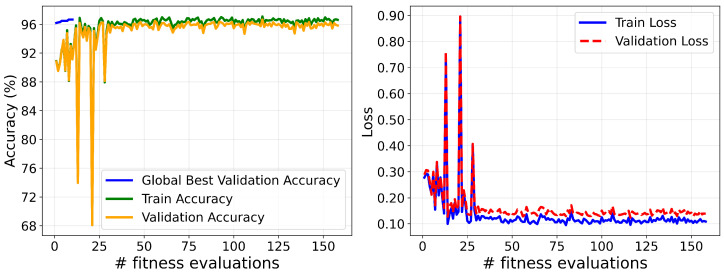
Accuracy and loss convergence for the proposed approach model.

The final model was trained using the Adam optimizer, with the optimal hyperparameters obtained through the GWO. The optimization process achieved a peak validation overall accuracy of 97% and a minimum validation loss of 0.12, reflecting a strong fit to both training and validation datasets. The corresponding convergence of accuracy and loss during training is illustrated in Figure 17.

The optimized proposed approach, the 1D-CNN–LSTM model, demonstrated computational efficiency, containing only 298,770 trainable parameters with a compact size of 1.14 MB. The training process was completed in approximately 1099 s (18 min) on an NVIDIA Tesla T4 GPU with 14.74 GB of memory. These results indicate that the proposed model is lightweight, resource-efficient, and feasible for deployment in practical healthcare applications.

The novelty of this research lies in the integration of 1D-CNN–LSTM with the GWO, which, unlike prior works, unifies deep feature extraction, temporal sequence learning, and metaheuristic-based hyperparameter tuning within a single framework [23,24,25,26,27]. While earlier studies employed 1D-CNN–LSTM without optimization, this work applies the GWO to automatically tune the hyperparameters of the hybrid 1D-CNN–LSTM model. Instead of relying on manual configuration, which is often time-consuming and suboptimal, the GWO offers an intelligent metaheuristic strategy to efficiently search for optimal hyperparameter settings, thereby enhancing the model’s overall accuracy and robustness. Other research has applied the GWO to alternative architectures such as an ANN or R-CNN [26,27]; however, this research utilized the complementary strengths of the 1D-CNN, which excels at extracting spatial and morphological features from ECG signals, and LSTM, which effectively captures temporal dependencies in heartbeat sequences. This proposed approach design, optimized through the GWO, enabled the proposed model to achieve superior performance across all arrhythmia classes. The results demonstrate that GWO-driven optimization of 1D-CNN–LSTM significantly enhances classification accuracy, reaching 97%. Furthermore, by benchmarking against both classical ML models (GBM and MLP) and DL baselines models, this research provides clear evidence that the proposed approach delivers superior and robust performance across all five AAMI arrhythmia classes.

To further evaluate the effectiveness of the proposed approach model (1D-CNN-LSTM) enhanced with the GWO, a comparison was performed with recent state-of-the-art studies that have addressed the classification of ECG arrhythmias using either DL or metaheuristic-based optimization methods in the MIT-BIH dataset. Table 12 summarizes the key characteristics and classification accuracies reported in these studies, in contrast to the results achieved in this work.

As shown in Table 12, the proposed model outperforms prior studies in terms of classification accuracy on the MIT-BIH dataset. The integration of a 1D-CNN–LSTM hybrid architecture with metaheuristic-based hyperparameter tuning via the GWO contributed to this improvement, underscoring the effectiveness of combining DL with nature-inspired optimization techniques for ECG arrhythmia detection. Furthermore, unlike previous models that often require millions of parameters and substantial storage, the optimized 1D-CNN–LSTM in this research contained only 298,770 trainable parameters (1.14 MB) and completed training in approximately 18 min, demonstrating computational efficiency and practical feasibility for real-world deployment.

Although the proposed approach 1D-CNN–LSTM model with the GWO achieved 97% overall accuracy, it is important to acknowledge that some recent works have reported accuracies above 98% [21,24,25]. However, these approaches often rely on complex attention mechanisms or image-based ECG representations, which increase computational cost. In contrast, our model balances strong accuracy with efficiency and simplicity, making it more practical for clinical deployment.

Several significant limitations should be noted even though the proposed 1D-CNN–LSTM model optimized using the GWO achieved improved performance. First, this research relies solely on the MIT-BIH dataset, which restricts the generalizability of the findings and may not fully capture the diversity of broader clinical populations. Second, class imbalance was addressed only through selective sampling, without comparisons to alternative approaches such as SMOTE, focal loss, or class weighting, which may provide different perspectives on performance. Third, statistical validation techniques, including k-fold cross-validation, confidence intervals, or hypothesis testing, were not performed due to computational constraints, which limits the ability to confirm the robustness of the reported accuracy. Finally, the model does not include explainability analysis. Methods such as Grad-CAM, saliency maps, or attention-based mechanisms could provide useful insights into the decision-making process of the model, thereby improving interpretability and clinical trust.

The proposed approach 1D-CNN–LSTM model optimized with the GWO demonstrates promising results for automated arrhythmia classification. In clinical practice, such a system could be integrated as a decision support tool to assist cardiologists and healthcare providers in the early detection of abnormal heart rhythms. For example, the model could be embedded in hospital ECG monitoring systems to provide real-time alerts for arrhythmic events. This would enable faster diagnosis, reduce the burden on physicians, and potentially improve patient outcomes through timely interventions.

However, several challenges must be addressed before clinical deployment. First, variations in ECG acquisition devices and patient populations may affect model performance, underscoring the need for validation on diverse datasets. Second, the interpretability of DL predictions remains limited, which may hinder trust among clinicians. Third, integration into existing healthcare infrastructure requires consideration of data privacy, regulatory approval, and user training. Addressing these challenges is essential to ensure the safe, reliable, and effective adoption of the model in real-world diagnostic workflows.

## 6. Conclusions and Future Work

Arrhythmia, or irregular heart rhythm, is a significant global health issue affecting millions worldwide and often leading to severe complications such as heart failure and stroke [47]. This growing prevalence underscores the critical need for precise and effective diagnostic methods to improve patient outcomes. Motivated by the importance of accurate arrhythmia detection, this work explores advanced ML and DL techniques to enhance ECG signal classification.

This work aimed to enhance classification accuracy in arrhythmia detection, addressing the critical need for reliable and precise diagnoses to support dependable clinical decision-making. To address this, a novel proposed approach was developed, integrating 1D-CNN and LSTM networks. Each model contributes unique strengths: 1D-CNNs are adept at identifying key spatial features within ECG data, while LSTMs capture the temporal patterns essential for analyzing sequential data like ECG signals.

The proposed appraoch model (1D-CNN–LSTM) was evaluated against individual models, including 1D-CNN, LSTM, and traditional ML algorithms such as GBM and MLP. The results demonstrated that the proposed system consistently outperformed these single models in diagnostic accuracy, precision, and reliability, achieving 97%. Specifically, the accuracy rates for the individual models were as follows: GBM achieved 91%, MLP scored 95%, LSTM reached 75%, and 1D-CNN obtained 92%.

To enhance the model’s accuracy and robustness, a series of preprocessing steps were applied to the ECG data. These steps included R-peak detection, normalization, sampling, and noise reduction via the DWT. These methods were essential for improving signal clarity and ensuring data quality for training and validation. The use of the MIT-BIH Arrhythmia Database provided a reliable basis for testing and validating these techniques.

Additionally, the hyperparameters of the proposed hybrid model were optimized using the GWO, a metaheuristic algorithm that mimics the social hierarchy and hunting behavior of gray wolves. This approach efficiently explored the hyperparameter space, replacing manual tuning and significantly improving the model’s performance and generalization ability.

The limitations of this research include its reliance on a single dataset, the restricted approach to handling class imbalance, the absence of statistical validation, and the lack of explainability features. To address these issues, future work will focus on validating the framework across additional datasets to improve generalizability and on exploring advanced imbalance handling methods such as SMOTE, focal loss, or class weighting. Statistical validation techniques, including k-fold cross-validation, confidence intervals, and hypothesis testing, will also be integrated to confirm the robustness of the findings. Furthermore, incorporating interpretability tools such as Grad-CAM, saliency maps, or attention-based visualization will strengthen clinical trust.

Future work will also involve exploring multi-label classification, as many patients may present with multiple co-morbid conditions rather than single arrhythmia types. Developing models capable of detecting multiple conditions simultaneously would better reflect clinical reality. Additionally, hybrid approaches that integrate DL for feature extraction with ML classifiers could achieve a balance between high accuracy and improved interpretability, making them more suitable for deployment in healthcare settings.

## Figures and Tables

**Table 1:** Arrhythmia classes according to the AAMI guidelines.

Class	Description
N	Normal beats
S	Supraventricular ectopic beats (abnormal beats originating above the ventricles)
V	Ventricular ectopic beats (abnormal beats originating in the ventricles)
F	Fusion of normal and ectopic beats
Q	Beats with uncertain classification

**Table 2:** Distribution of training and testing samples (80/20 split).

Classes	Training Samples	Testing Samples	Total Samples
N	22,297	5575	27,872
S	1953	488	2441
V	5472	1368	6840
F	629	158	787
Q	3085	771	3856
Total	33,436	8360	41,796

**Table 3:** Architecture details of 1D-CNN.

Layers	Channels	Filters	Kernel Size	Stride	Padding
Cov 1D	1	256	8	5	1
ReLU					
Dropout: 0.15					
MaxPool	-	2	2	-	-
Cov 1D	256	128	7	4	1
ReLU					
Dropout: 0.1					
MaxPool	-	2	2	-	-
Flatten	-	-	-	-	-
Dense: Units: 128					
SoftMax: Output: 5					

**Table 4:** LSTM architecture details.

Layers	Input Size	Hidden Size	Batch First
LSTM	1	64	True
LSTM	64	32	True
Dropout: 0.4			
LSTM	32	16	True
Fully Connected: Units = 16			
Softmax: Output = 5			

**Table 5:** Hyperparameter ranges used in GWO optimization.

Hyperparameter	Search Range
Number of Filters	[8, 320]
Kernel Size	[3, 7]
Stride Size	[2, 8]
LSTM Units	[20, 256]
Dropout Rate	[0.001, 0.6]

**Table 6:** Evaluation metrics for ML and DL models.

Evaluation Metric	1D-CNN	LSTM	GBM	MLP	Proposed Approach
Precision (%)	80	62	91	93	93
Recall (%)	90	68	74	84	88
F1 Score (%)	84	64	80	88	90
Overall Accuracy (%)	92	75	91	95	97

**Table 7:** Evaluation metrics for the GBM across classes.

Evaluation Metric	N	S	V	F	Q	Average
Precision (%)	90	83	95	88	99	91
Recall (%)	99	45	81	49	94	74
F1 Score (%)	94	58	88	63	96	80
Overall Accuracy (%)	91					

**Table 8:** Evaluation metrics for the MLP model across classes.

Evaluation Metric	N	S	V	F	Q	Average
Precision (%)	95	91	94	86	98	93
Recall (%)	99	72	90	59	98	84
F1 Score (%)	97	81	92	70	98	88
Overall Accuracy (%)	95					

**Table 9:** Evaluation metrics for the 1D-CNN model across classes.

Evaluation Metric	N	S	V	F	Q	Average
Precision (%)	97	71	86	47	99	80
Recall (%)	92	84	91	83	100	90
F1 Score (%)	95	77	89	60	99	84
Overall Accuracy (%)	92					

**Table 10:** Evaluation metrics for the LSTM model across classes.

Evaluation Metric	N	S	V	F	Q	Average
Precision (%)	92	35	80	13	90	62
Recall (%)	83	56	75	39	89	68
F1 Score (%)	87	43	78	20	90	64
Overall Accuracy (%)	75					

**Table 11:** Evaluation metrics for the proposed approach 1D-CNN–LSTM model across classes.

Evaluation Metric	N	S	V	F	Q	Average
Precision (%)	96	88	96	84	99	93
Recall (%)	99	81	92	71	98	88
F1 Score (%)	98	84	94	77	99	90
Overall Accuracy (%)	97					

**Table 12:** Comparison of the proposed approach model with related works.

Study	Model/Approach	Key Techniques	Accuracy
Mishra & Agrawal (2020) [27]	Artificial Neural Network (ANN)	GWO for tuning	94%
Mirdan (2025) [45]	Attention-based Deep Network	Class imbalance handling, deep attention	94%
Zhao et al. (2020) [46]	Deep CNN	Wavelet transform for feature enhancement	95%
**This Research**	CNN + LSTM Hybrid Model	GWO for hyperparameter tuning	**97%**

## Data Availability

The ECG data used in this study are publicly available from the MIT-BIH Arrhythmia Database.
